# Normalization of adiponectin concentrations by leptin replacement in *ob/ob* mice is accompanied by reductions in systemic oxidative stress and inflammation

**DOI:** 10.1038/s41598-017-02848-0

**Published:** 2017-06-05

**Authors:** Gema Frühbeck, Victoria Catalán, Amaia Rodríguez, Beatriz Ramírez, Sara Becerril, Piero Portincasa, Javier Gómez-Ambrosi

**Affiliations:** 1 Metabolic Research Laboratory, Clínica Universidad de Navarra, Pamplona, Spain; 2 CIBER Fisiopatología de la Obesidad y Nutrición (CIBEROBN), Instituto de Salud Carlos III, Pamplona, Spain; 3 Obesity and Adipobiology Group, Instituto de Investigación Sanitaria de Navarra (IdiSNA), Pamplona, Spain; 4 Department of Endocrinology & Nutrition, Clínica Universidad de Navarra, Pamplona, Spain; 5 Clinica Medica “A. Murri”, Department of Biomedical Sciences and Human Oncology, University of Bari Medical School, Policlinico Hospital, Bari, Italy

## Abstract

The circulating concentrations of adiponectin, an antidiabetic adipokine, have been shown to be reduced in obesity, in relation to an increase in inflammation. The aim of the present work was to assess the effect of leptin replacement on adiponectin levels and expression as well as on markers of oxidative stress and inflammation in leptin-deficient *ob/ob* mice. Twelve-week-old male mice (n = 7–10 per group) were treated with either saline (wild type and *ob/ob* mice) or leptin (*ob/ob* mice) for 18 days. A third group of *ob/ob* mice was treated with saline and pair-fed to the amount of food consumed by the leptin-treated group. Leptin replacement restored values of adiponectin (*P* < 0.001), reduced circulating 8-isoprostane and serum amyloid A (SAA) levels (*P* < 0.05 for both), and significantly downregulated the increased gene expression of osteopontin (*Spp1*, *P* < 0.05), *Saa3* (*P* < 0.05), *Cd68* (*P* < 0.01), *Il6* (*P* < 0.01) and NADPH oxidase (*Nox1* and *Nox2*, *P* < 0.01) in the perirenal WAT and *Spp1* (*P* < 0.05) in the liver of *ob/ob* mice. In cultured adipocytes from *ob/ob* mice, leptin increased (*P* < 0.05) the mRNA expression and secretion of adiponectin. We concluded that circulating concentrations of adiponectin are positively regulated by leptin and ameliorate obesity-associated oxidative stress and inflammation in mice.

## Introduction

Over the last decades, changes in lifestyle have caused a progressive increase in the incidence of obesity, being one of the most prevalent metabolic disorders^1, 2^. Excess adiposity favors the development of cardiometabolic alterations such as type 2 diabetes (T2D), cardiovascular disease, dyslipidemia, steatohepatitis and cancer^1^. Given the increasing prevalence of obesity and its comorbidities, further research to unravel the metabolic mechanisms involved in its etiopathogenesis is necessary^3^.

Adipose tissue secretes a wide variety of biologically active molecules representing an extremely active endocrine organ^3^. These secreted proteins, collectively called adipokines, such as leptin and adiponectin, are known to be involved in the pathophysiological link between increased adiposity and cardiometabolic abnormalities^3^. Leptin is a 16 kDa protein primarily produced by adipose tissue in proportion to the amount of body adiposity functioning as a lipostat signal informing the hypothalamus on the size of fat deposits in the body^3^. Circulating leptin concentrations are higher in obese individuals, which has led to the concept of leptin resistance^4^. Besides its regulatory function of energy homeostasis, leptin is involved in the regulation of neuroendocrine function, haematopoiesis, angiogenesis, and reproduction, among others^5^.

Adiponectin is another important adipokine of 30 kDa expressed almost exclusively in adipose tissue^6^. Plasma adiponectin concentrations are decreased in obese patients as well as in people with cardiovascular disease^7^. Adiponectin increases insulin sensitivity and prevents lipid accumulation in skeletal muscle and liver, stimulating fatty acid oxidation. Adiponectin has been also reported to exert anti-inflammatory actions^8^. The beneficial effects of adiponectin are mediated primarily via the action of two receptors, AdipoR1 and AdipoR2 which exhibit overlapping and distinct effects^9^.

Obesity may induce systemic oxidative stress in relation with and altered adipokine secretion^10^. Oxidative stress plays a critical role in the development of obesity associated comorbidities favoring the appearance of T2D, hypertension, liver steatosis and atherosclerosis^11^. Moreover, systemic and local inflammation is also increased in obesity leading to an increased risk of the development of a wide number of comorbidities^12, 13^. Leptin-deficient *ob/ob* mice exhibit massive obesity, dyslipidemia and insulin resistance. These mice have been proposed as a model of the metabolic syndrome since they exhibit many of the pathophysiologic alterations most frequently associated with obesity, including increased oxidative stress^14–16^ and elevated systemic inflammation^14, 17^.

The effect of leptin on circulating adiponectin concentrations has been previously studied with contradictory results. In this sense, leptin-deficient *ob/ob* mice, in which leptin’s effect are absent, exhibit either decreased^18, 19^, increased^20, 21^, or unchanged^22^ adiponectin circulating concentrations. Moreover, many previous studies have used supraphysiological or even pharmacological doses of leptin^18, 23^ that may overestimate its effects or, alternatively, exert no effect since it has been reported that pharmacological doses of leptin may lose its effect due to saturation or downregulation of receptors^24^ or even elicit opposite effects to those observed after the administration of a low leptin dose around the physiological level^25^.

The aim of the present work was to assess the effect of exogenous administration of leptin at a low dose on circulating concentrations and adipose tissue expression of adiponectin in leptin-deficient *ob/ob* mice as well as its impact on systemic and adipose tissue and liver inflammation and oxidative stress.

## Results

### Leptin administration restores adiponectin concentrations in *ob/ob* mice in association with a decrease in oxidative stress and markers of inflammation

**Figure 1:**
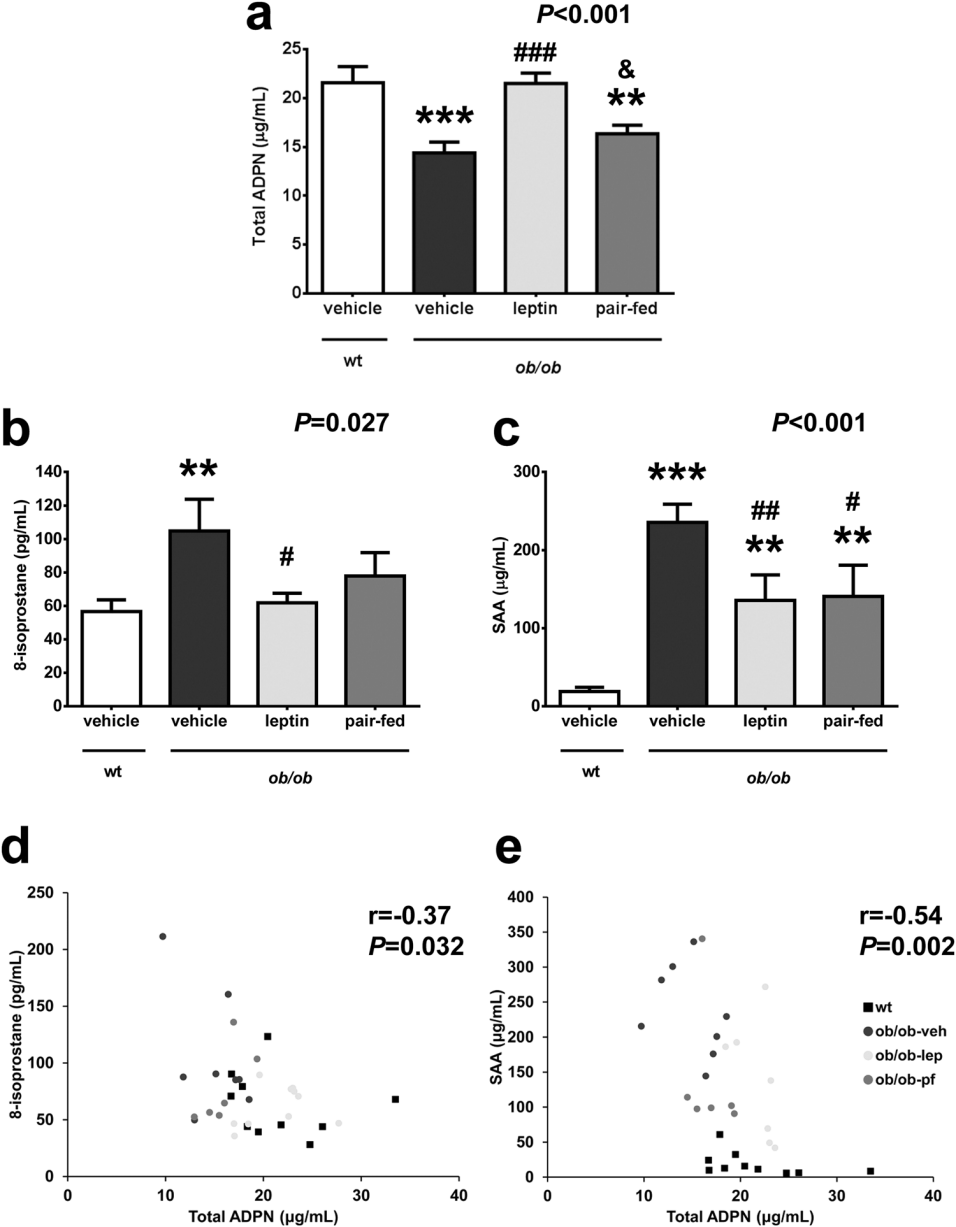
Leptin replacement increases total adiponectin (ADPN) concentrations, which were reduced in *ob/ob* mice, reducing systemic inflammation and oxidative stress. Serum levels of total adiponectin (**a**), 8-isoprostane (**b**) and serum amyloid A (SAA) (**c**) in wild type (wt) and *ob/ob* mice receiving vehicle, leptin or receiving vehicle and pair-fed to the leptin group. Data presented as mean ± SEM. Differences between groups were analyzed by one way ANOVA followed by LSD test. **P < 0.01 and ***P < 0.001 *vs* wt; ^#^ *P* < 0.05, ^##^ *P* < 0.01 and ^###^ *P* < 0.001 *vs ob/ob* treated with vehicle; ^&^ *P* < 0.05 *vs ob/ob* treated with leptin. Scatter diagrams showing the negative correlation found between the circulating concentrations of total adiponectin and the levels of 8-isoprostane (**d**) and SAA (**e**). Pearson’s correlation coefficient and *P* values are indicated. n = 6–10 per group.

As expected, leptin-deficient obese *ob/ob* mice showed increased body weight and food intake as well as higher serum glucose, insulin, triglyceride and total cholesterol concentrations as compared to wt mice (Table 1). Serum concentrations of total adiponectin were significantly reduced in *ob/ob* mice as compared to wt littermates (14.4 ± 1.1 *vs* 21.6 ± 1.7 μg/mL; *P* < 0.001, Fig. 1a). Leptin treatment significantly reduced body weight, food intake as well as serum variables after 18 days of treatment (Table 1). Serum total adiponectin levels were normalized after leptin replacement (21.5 ± 1.1 μg/mL; *P* < 0.001 *vs* untreated *ob/ob*) while the PF group exhibited very similar levels to vehicle-treated *ob/ob* mice (16.3 ± 0.9 μg/mL; *P* < 0.05 *vs* leptin-treated *ob/ob* mice). Systemic oxidative stress was significantly higher in *ob/ob* mice, as evidenced by 8-isoprostane levels (105 ± 19 *vs* 57 ± 7 pg/mL; *P* < 0.01) with leptin administration normalizing its levels (62 ± 6 pg/mL; *P* < 0.05 *vs* untreated *ob/ob*). Pair-fed *ob/ob* mice exhibited an improvement in 8-isoprostane concentrations (78 ± 14 pg/mL), showing a half-way phenotype between leptin- and saline-treated *ob/ob* mice (Fig. 1b). Leptin administration significantly reduced circulating concentrations of the acute phase reactant serum amyloid A (SAA), which were dramatically increased in the *ob/ob* mice, to a similar extent than pair-feeding (wt: 19 ± 5; *ob/ob*-vehicle: 236 ± 23; *ob/ob*-leptin: 136 ± 33; *ob/ob* pair-fed: 141 ± 40 μg/mL; *P* < 0.001, Fig. 1c). Serum total adiponectin concentrations were negatively correlated with the circulating levels of 8-isoprostane (r = −0.37, *P* = 0.032; Fig. 1d) and SAA (r = −0.54, *P* = 0.002; Fig. 1e).

**Table 1:** Body weight, food intake and serum analysis in *ob/ob* mice. Effect of leptin replacement.

	Wild type	*ob/ob*	*ob/ob* + Leptin	*ob/ob* Pair-Fed
Body weight (g)	23.5 ± 0.3	47.8 ± 0.7***	43.1 ± 0.6***^,###^	44.4 ± 0.8***^,###^
Food intake (g)	3.2 ± 0.2	5.5 ± 0.5***	4.6 ± 0.6***^,###^	4.7 ± 0.6***^,###^
Glucose (mg/dL)	72 ± 6	295 ± 13***	247 ± 18***^,#^	280 ± 17***
Insulin (pmol/L)	0.2 ± 0.1	14.2 ± 1.1***	6.7 ± 1.4**^,###^	16.4 ± 1.8***^,&&&^
HOMA	1.2 ± 0.3	246.2 ± 18.0***	110.2 ± 1.4**^,###^	279.8 ± 40.8***^,&&&^
Triglycerides (mg/dL)	97 ± 6	136 ± 14**	106 ± 3^#^	107 ± 16
Cholesterol (mg/dL)	63 ± 9	180 ± 10***	99 ± 11*^,###^	136 ± 13***^,##,&&^

HOMA, homeostatic model assessment. Data presented as mean ± SEM. Differences were analyzed by ANOVA followed by LSD tests. *P < 0.05, **P < 0.01 and ***P < 0.001 *vs* wild type; ^#^ *P* < 0.05, ^##^ *P* < 0.01 and ^###^ *P* < 0.001 *vs ob/ob*; ^&&^ *P* < 0.01 and ^&&&^ *P* < 0.001 *vs ob/ob* + leptin. n = 6–10 per group.

### Leptin raises adiponectin concentrations increasing its expression and secretion in adipose tissue

**Figure 2:**
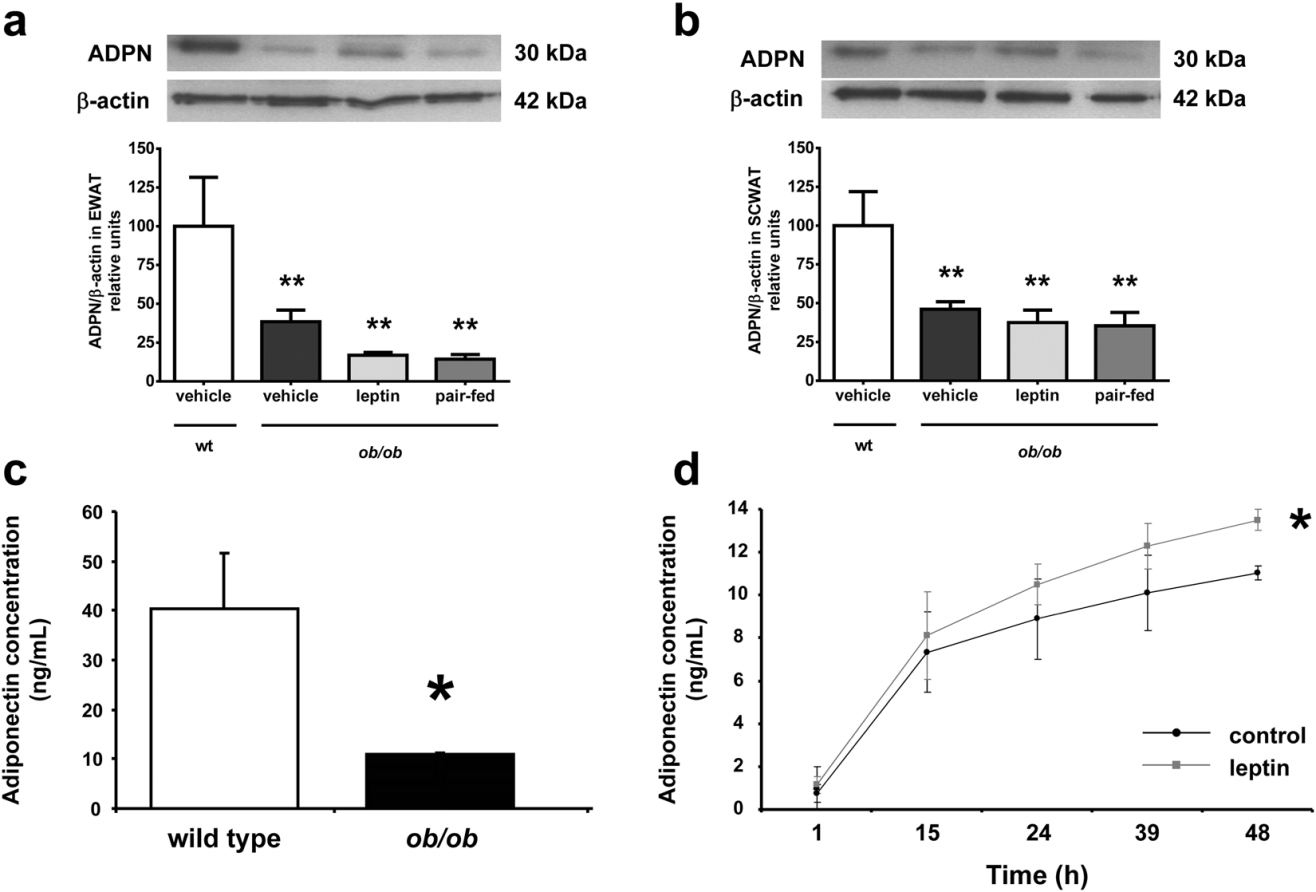
Protein amount of adiponectin is decreased in white adipose tissue of *ob/ob* mice and leptin stimulates its secretion from adipocytes. Protein expression levels of total adiponectin (ADPN) in mouse epididymal (EWAT, **a**) and subcutaneous (SCWAT, **b**) adipose tissues from wild type (wt) and *ob/ob* mice receiving vehicle, leptin or receiving vehicle and pair-fed to the leptin group. The blot densitometry data were normalized with β-actin values (upper panels). Values are the mean ± SEM. The expression of ADPN in the wt group was assumed to be 100. Differences between groups were analyzed by ANOVA followed by LSD tests. **P < 0.01 *vs* wt. (**c**) Concentrations of adiponectin in secreted media from EWAT adipocytes differentiated from wt and *ob/ob* mice after 48 h of culture (n = 3–7 mice). *P < 0.05 *vs* wt by two-tailed unpaired Student’s *t* test. (**d**) Effect of leptin at a concentration of 10^−8^ mol/L on the amount of adiponectin in the secreted media in adipocytes differentiated from *ob/ob* mice up to 48 h of culture. The effect of treatment was analyzed by unpaired Student’s *t* tests. *P < 0.05 *vs* unstimulated adipocytes.

**Figure 3:**
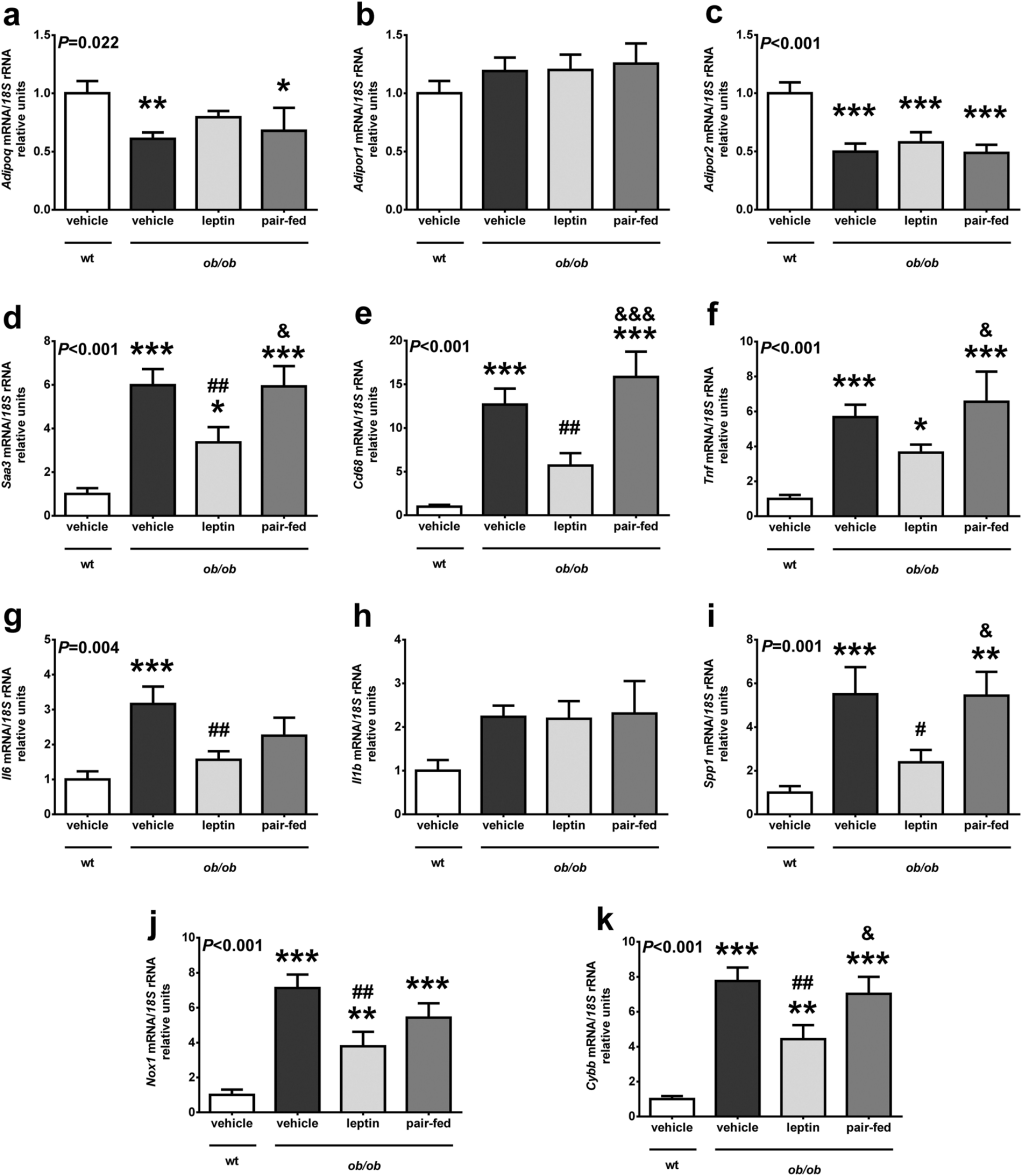
Leptin replacement in *ob/ob* mice regulates the expression of genes involved in inflammation and oxidative stress in adipose tissue. Gene expression levels of *Adipoq* (**a**), *Adipor1* (**b**), *Adipor2* (**c**), *Saa3* (**d**), *Cd68* (**e**), *Tnf* (**f**), *Il6 (* ***g*** *), Il1b (* ***h*** *), Spp1* (**i**), *Nox1* (**j**) and *Cybb* (**k**) in perirenal adipose tissue in wild type (wt) and *ob/ob* mice receiving vehicle, leptin or receiving vehicle and pair-fed to the leptin group. Data presented as mean ± SEM of 6–10 animals. Differences between groups were analyzed by one way ANOVA followed by LSD test. *P < 0.05, **P < 0.01 and ***P < 0.001 *vs* wt; ^#^ *P* < 0.05 and ^##^ *P* < 0.01 *vs ob/ob* treated with vehicle; ^&^ *P* < 0.05 and ^&&&^ *P* < 0.001 *vs ob/ob* treated with leptin.

The amount of adiponectin protein in EWAT and SCWAT was significantly reduced in all *ob/ob* groups as compared to wt mice (*P* < 0.01) with leptin or pair-feeding exerting no effect (Fig. 2a and b). Adiponectin released to the media was significantly lower (*P* < 0.05) in EWAT differentiated adipocytes from *ob/ob* mice than that from wt mice after 48 h of culture (Fig. 2c). After 48 h of culture leptin significantly upregulated (*P* < 0.05) adiponectin secretion to the media in differentiated adipocytes from *ob/ob* mice (Fig. 2d). *Adipoq* mRNA expression was downregulated in *ob/ob* mice as compared to wt (*P* < 0.01). The *in vitro* increased secretion and the absence of changes in the amount of protein in WAT after *in vivo* leptin administration was accompanied by a leptin-induced upregulation in adiponectin mRNA expression in the *ob/ob* mice, showing no difference with the wt mice (Fig. 3a).

### Leptin reduces the expression of genes involved in inflammation and oxidative stress in adipose tissue

No effect of leptin on the expression of adiponectin receptors in adipose tissue was observed (Fig. 3b and c). Adipose tissue from *ob/ob* exhibited a dramatic increase (2.5- to 12-fold, *P* < 0.001) in the expression of genes involved in inflammation. Leptin administration significantly reduced (*P* < 0.05) the expression of *Saa3*, *Cd68, Il6* and *Spp1* in *ob/ob* as compared to vehicle-treated littermates (Fig. 3d,e,g and i). *Tnf* mRNA showed the same trend, although the differences with the vehicle group were only marginal (*P* = 0.073) (Fig. 3f), while no changes were observed in the expression levels of *Il1b* (Fig. 3h). In a similar way, adipose tissue from leptin-deficient *ob/ob* mice exhibited an increase (7- to 8-fold, *P* < 0.001) in the expression of genes related to oxidative stress. Leptin replacement significantly reduced (*P* < 0.01) the expression of *Nox1* and *Cybb* in *ob/ob* as compared to vehicle-treated *ob/ob* mice (Fig. 3j and k)

### Leptin ameliorates liver steatosis and reduces the expression of genes involved in inflammation in the liver

**Figure 4:**
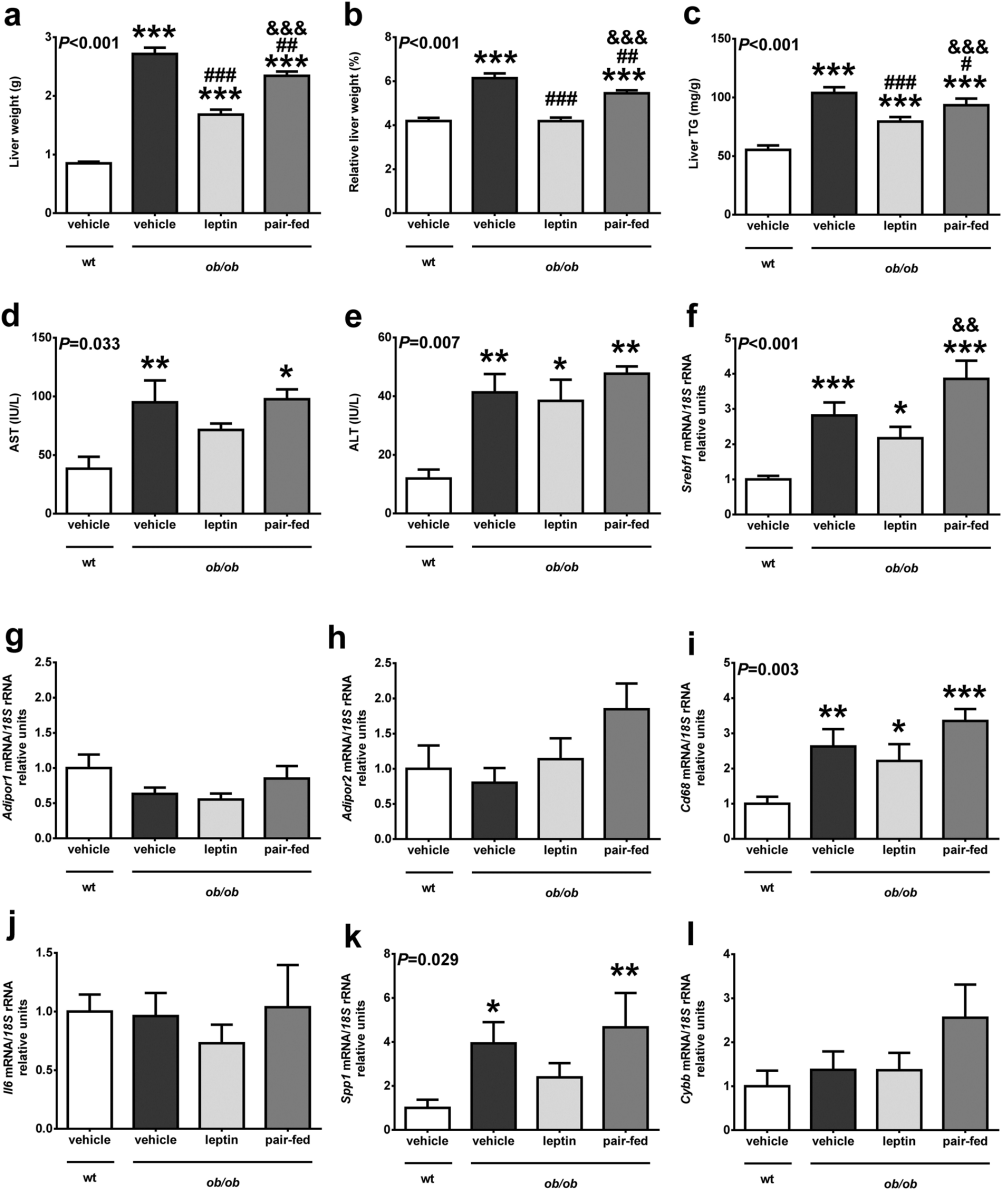
Leptin replacement in *ob/ob* mice reduces liver steatosis and regulates the expression of genes involved in inflammation. Liver weight (**a**), relative liver weight (**b**), triglyceride content in the liver (**c**) and serum AST (**d**) and ALT (**e**) levels of the animals from the different experimental groups. Mean ± SEM of 7–10 animals. (**f**) Expression of the lipogenic gene *Srebf1* in the liver. Gene expression levels of *Adipor1* (**g**), *Adipor2* (**h**), *Cd68* (**i**), *Il6* (**j**), *Spp1* (**k**) and *Cybb* (**l**) in the liver in wild type (wt) and *ob/ob* mice receiving vehicle, leptin or receiving vehicle and pair-fed to the leptin group. Data presented as mean ± SEM of 6–10 animals. Differences between groups were analyzed by one way ANOVA followed by LSD test. *P < 0.05, **P < 0.01 and ***P < 0.001 *vs* wt; ^#^ *P* < 0.05, ^##^ *P* < 0.01 and ^###^ *P* < 0.001 *vs ob/ob* treated with vehicle; ^&&^ *P* < 0.01 and ^&&&^ *P* < 0.001 *vs ob/ob* treated with leptin.

Liver weight was higher in *ob/ob* mice and significantly reduced (*P* < 0.001 as compared to *ob/ob* mice receiving vehicle) by leptin treatment (Fig. 4a), showing no differences in relative terms with wt mice (Fig. 4b). Analysis of intrahepatic TG content showed elevated TG levels in *ob/ob* mice and that leptin replacement significantly (*P* < 0.001) reduced them (Fig. 4c). This reduction in TG was accompanied by a decrease in AST levels in the leptin-treated group, showing no statistical differences with the wt group, that was not observed after pair-feeding (Fig. 4d). No effect of leptin was found on ALT levels (Fig. 4e). Pair-fed *ob/ob* mice exhibited a halfway liver phenotype between leptin- and saline-treated *ob/ob* mice (Fig. 4a–c). Lack of leptin was associated with an increase in the mRNA levels of the lipogenic transcription factor *Srebf1*, whose expression was slightly reduced after leptin treatment, showing significant differences only with the pair-fed group (Fig. 4f). As observed in adipose tissue, no effect in the expression of adiponectin receptor after leptin administration was shown in the liver (Fig. 4g and h). Increased expression of genes involved in inflammation such as *Cd68* and *Spp1* in *ob/ob* mice was observed, which was normalized after leptin administration only in the case of *Spp1* (Fig. 4i and k). No changes were observed in the hepatic expression levels of *Il6* and *Cybb* (Fig. 4j and l).

## Discussion

The major findings of the present study are that leptin replacement: (1) restored serum concentrations of adiponectin, which were decreased in *ob/ob* mice, through the increase in its expression and secretion, (2) reduced the levels of markers of oxidative stress, such as 8-isoprostane, and inflammation, such as SAA levels, and (3) downregulated the increased expression of genes involved in inflammation such as *Spp1* (Opn), *Cd68*, *Il6* and *Saa3* in the WAT and *Spp1* in the liver, and oxidative stress, such as NADPH oxidase (*Nox1* and *Cybb*) in the WAT of *ob/ob* mice.

The analysis of the serum concentrations of adiponectin in *ob/ob* mice has produced discrepant results, with reported lower^18, 19^, higher^20, 21^, or unchanged^22^ levels. Data from the present study and from previous studies of our group^19^ clearly show that *ob/ob* mice exhibit reduced serum adiponectin levels. One of the factors that may explain these discrepancies is the genetic background of the *ob/ob* mice, which has been shown to strongly influence the metabolic impact of the leptin deficiency^26^, although most of the reported studies have used *ob/ob* mice on a B6 background^27^. Other factors such as the age of the animals, diet or fasting conditions offer alternative explanations.

Leptin replacement restored serum adiponectin to normal levels, in agreement with a previous study with a higher subcutaneous dose of leptin in a shorter period of time (6 d)^18^. This effect was beyond the inhibitory action of leptin on food intake, since pair-fed animals showed adiponectin levels closer to the vehicle-treated than to the leptin-treated *ob/ob* mice. This effect is in contrast to another work where central leptin administration reduced adiponectin concentrations^28^. Further studies might elucidate whether leptin regulates central or peripherally adiponectin levels differentially.

Leptin administration induced an increase in *Adipoq* mRNA expression in WAT. Previous studies have shown lower adiponectin mRNA expression in leptin-deficient *ob/ob* and leptin receptor-deficient *db/db* mice^29–31^, as well as in obese Zucker *fa/fa* rats lacking functional leptin receptors^32^, and that leptin significantly increases *Adipoq* expression in WAT^18, 23, 33, 34^ and in the heart^35^. Interestingly, we found lower amounts of adiponectin protein in WAT of *ob/ob* mice consistent with other studies^30, 36, 37^, but leptin replacement failed to normalize its levels. This *in vivo* observation prompted us to hypothesize that the restored circulating levels of adiponectin with an increase in *Adipoq* mRNA expression but without changes in the protein levels in WAT is due to an increased expression and secretion in WAT. Our *in vitro* studies evidenced a reduced adiponectin secretion in adipocytes from *ob/ob* mice and that leptin significantly increased adiponectin secretion after 48 h of exposure confirming our hypothesis. A recent study did not observe the same stimulatory effect of leptin in adiponectin secretion in human white adipocytes, but the authors studied leptin effects only after 24 h of treatment^38^, a period of time in which we also did not observe a significant effect of leptin. It seems that more prolonged periods of time (up to 48 h) are needed to observe such an effect. We conclude that leptin regulates adiponectin concentrations stimulating its expression and secretion from WAT.

The restoration of adiponectin concentrations after leptin replacement in *ob/ob* mice was accompanied by a decrease in systemic inflammation and oxidative stress. Since obese *ob/ob* mice exhibit increased inflammation and oxidative stress compared with their lean littermates^14^, we aimed to study systemic levels of 8-isoprostane and SAA as well as genes involved in inflammatory and oxidative stress in WAT and liver of obese *ob/ob* mice. Oxidative stress is defined as an imbalance in the redox state resulting in an increased production of reactive oxygen species (ROS), ultimately leading to oxidative damage of cellular components. Oxidative stress was increased in *ob/ob* mice as evidenced by the elevated levels of 8-isoprostane together with the increase in the expression of the NADPH oxidase subunits *Nox1* and *Nox2* (*Cybb*) in WAT, which encode the major enzymes generating ROS^10^. These observations are in agreement with a large number of studies related to increased serum oxidative stress in obesity both in animal models and humans^10, 11, 39^. Leptin administration restored 8-isoprostane levels to normality and significantly reduced the expression of *Nox1* and *Cybb*. However, the relationship between leptin and oxidative stress has not been fully elucidated. Leptin stimulates *in vitro* ROS production in different cell types^40, 41^ and systemic oxidative stress in animal models^42^, suggesting a stimulatory role of leptin. On the contrary, oxidative stress is increased in obese Zucker *fa/fa* rats^39^, while leptin administration reduces the oxidative stress in different cellular^43^ or rodent models^44, 45^. In the present study, exogenous administration of leptin to *ob/ob* mice repressed the increased expression of *Nox1* and *Cybb* in WAT. These findings are in line with previous observations showing that leptin restores the defective antioxidant enzyme capacity in serum of leptin-deficient mice^46^ and humans^47^, but disagree with the reported increased p47phox (*Ncf1*) expression after leptin replacement in the livers of *ob/ob* mice^48^. It is possible that the much higher leptin dose used in the latter study explains the discrepant findings. Noteworthy, another study has reported *in vitro* a protective effect of leptin against ethanol-induced oxidative stress^49^ and our group has evidenced that leptin replacement reduces the expression of genes related to oxidative stress in the skeletal muscle of *ob/ob* mice^14^.

Acute-phase reactants have been suggested to contribute to the maintenance of the obesity-associated low-grade chronic inflammation^50, 51^. Interestingly, our study provides evidence that the acute-phase response was increased in *ob/ob* mice as evidenced by the increased serum concentrations of SAA, which were counteracted, at least in part, by exogenous leptin administration. Saa3 has been involved in an inflammatory paracrine loop between adipocytes and activated macrophages in mice, being proposed as an index of the number of activated macrophages monitoring the adipose tissue inflammatory state^52^. Tnf has been proposed as one of the major stimulators of adipose tissue production of Saa3 in this loop. However, the reduction of *Tnf* expression in WAT after leptin replacement in our study was only marginal, which suggests that the downregulation of other proinflammatory cytokines may be involved in this effect^53, 54^. Leptin administration reduced the elevated gene expression of *Saa3*, *Cd68* and Opn (*Spp1*) in WAT and *Spp1* in the liver, which are upregulated in obesity-associated inflammation in mice and humans^51, 55, 56^. A proinflammatory role has been attributed to leptin^57^. However, the consideration of leptin as a proinflammatory factor derives mostly from *in vitro* studies and may be in relation to its proliferative effect and its ability to stimulate the metabolism^58^. In this sense, leptin administration at physiological or pharmacological levels does not modify circulating inflammatory marker levels in humans^59, 60^. Taken together, our data suggest that leptin prevent the obesity-associated inflammatory state and the increased oxidative stress in leptin-deficient *ob/ob* mice. The stimulatory effect of leptin on circulating adiponectin levels in *ob/ob* mice could be involved in the amelioration in oxidative stress and inflammation observed in these animals, since a negative correlation between adiponectin and 8-isoprostane and SAA after leptin replacement was found. An increase in the expression of adiponectin receptors *Adipor1* and *Adipor2*, which were reduced in *ob/ob* in agreement with a previous work^37^, is unlikely to mediate these improvements, since their expression was unaffected after leptin administration.

Systemic concentrations of 8-isoprostane and SAA was reduced in pair-fed *ob/ob* mice (although not reaching statistical significance in the case of 8-isoprostane), showing that weight loss contributes to the improvement in oxidative stress and inflammation as previously reported^11, 51^. However, the effect of leptin on the expression of genes involved in inflammation and oxidative stress in adipose tissue and liver was beyond the leptin-induced inhibitory effect on food intake, evidencing a direct positive effect of leptin at the dose used. Similar findings were previously reported by our group in the skeletal muscle^14^. The effect of leptin on the expression of these genes in other organs merits further research.

The leptin-induced reduction in the expression of proinflammatory genes in the liver was accompanied by a significant reduction in TG accumulation and serum AST levels. This observation suggests that leptin replacement in *ob/ob* mice, even at the low dose employed, exerts anti-steatotic effects in the liver in agreement with previous reports from our group^61^ and others^62^ using higher doses of leptin. Our data point out that the reduction in inflammation and oxidative stress may contribute, together with the reduction in lipogenesis and increase in fatty-acid oxidation^63^, to the improvement of hepatic function after leptin replacement.

In summary, obesity is accompanied by a chronic pro-inflammatory state and increased oxidative stress. Findings of our study provide evidence that systemic oxidative stress and inflammation, together with the elevated expression of genes involved in oxidative stress and inflammation of *ob/ob* mice are reversed by leptin replacement. A rise in adiponectin concentrations seems to be involved in the metabolic improvement observed. The translational value of the present study is that subjects with adipose tissue dysfunction, characterized by a lower secretion of adiponectin in relation to leptin levels, may have increased systemic oxidative stress and inflammation. Therefore, therapeutic tools targeted to improve this dysfunctional adipokine secretion pattern in adipose tissue may render a lower oxidative and inflammatory profile and, consequently, a lower cardiometabolic risk.

## Materials and Methods

### Animals and treatments

Wild type (wt, C57BL/6) and leptin- deficient (B6.Cg-Lep^ob^/J, *ob/ob*, JAX™ Mice Stock Number 000632) mice were purchased from Charles River (Saint-Germain-sur-l′Arbresle, France). Mice were housed at an ambient temperature of 22 ± 2 °C on a 12:12 h light-dark cycle (lights on at 08:00 h) under pathogen-free conditions and given a standard laboratory diet (Rodent Toxicology Diet, B&K Universal Ltd., Hull, UK) and tap water *ad libitum*. Twelve-week-old male mice were treated with saline or leptin (0.1 mg/kg body weight) i.p. twice daily for 18 days; recombinant mouse leptin was purchased from PeproTech EC Ltd. (London, UK). In addition, another group of *ob/ob* mice was treated with saline and pair-fed to the amount of food consumed by the leptin-treated group in order to dissociate the well-known appetite reducing effect of leptin from other effects. Mice were killed by CO_2_ inhalation. Epididymal white adipose tissue (EWAT), subcutaneous WAT (SCWAT) and perirenal WAT (PRWAT) were snap-frozen in liquid nitrogen and stored at −80 °C until extraction of RNA. Plasma was separated and stored at −80 °C for subsequent measurements. All experimental procedures conformed to the European Guidelines for the Care and Use of Laboratory Animals and the study was approved by the Ethical Committee for Animal Experimentation of the University of Navarra.

### Serum measurements

Blood samples were collected by cardiac puncture. Serum glucose was determined by using a Blood Glucose Meter (Ascensia ELITE^®^, Bayer, Barcelona, Spain). Serum insulin was measured by means of an Ultra Sensitive Rat Insulin ELISA kit using mouse insulin as standard (Crystal Chem Inc., Chicago, IL, USA). Intra-and inter-assay coefficients of variation (CV) were 5.5% and 4.8%, respectively. Serum aspartate aminotransferase (AST) and alanine aminotransferase (ALT) were measured by enzymatic tests (Infinity™ AST or ALT Liquid Stable Reagent Thermo Fisher Scientific, Waltham, MA, USA). Serum adiponectin was quantified by ELISA (Biovendor, Modrice, Czech Republic). Intra-and inter-assay CV were 4.2% and 5.9%, respectively. Circulating levels of 8-isoprostane (8-iso PGF2a), a proposed marker of antioxidant deficiency and oxidative stress, were measured by an immunoenzymatic assay (Cayman Chemicals, Ann Arbor, MI, USA). SAA concentrations were assessed using an ELISA kit (BioSource International Inc., Camarillo, CA, USA). Intra-and inter-assay CV were 5.3% and 8.1%, respectively.

### RNA extraction and Real-Time PCR

Total RNA was extracted from 100 mg of PRWAT samples by homogenization with an ULTRA-TURRAX^®^ T 25 basic (IKA^®^ Werke GmbH, Staufen, Germany) using TRIzol reagent (Invitrogen, Carlsbad, CA, USA) and the RNeasy Lipid Tissue Mini Kit (Qiagen, Valencia, CA, USA). The RNA concentration was determined from absorbance at 260 nm. Transcript levels were quantified by Real-Time PCR (7300 Real-Time PCR System, Applied Biosystem, Foster City, CA, USA). Primers and probes (Sigma-Aldrich, Madrid, Spain) were designed using the software Primer Express 2.0 (Applied Biosystems). Primers and probes used to amplify the cDNA are described in Table 2. The cDNA was amplified at the following conditions: 95 °C for 10 min, followed by 45 cycles of 15 s at 95 °C and 1 min at 59 °C, using the TaqMan^®^ Universal PCR Master Mix (Applied Biosystems). The primer and probe concentrations for gene amplification were 300 nmol/L and 200 nmol/L, respectively. All results were normalized to the levels of *18 S* rRNA (Applied Biosystems) and relative quantification was calculated using the ΔΔCt formula^64^. Relative mRNA expression was expressed as fold expression over the calibrator sample (average of gene expression corresponding to the wt group). All samples were run in triplicate and the average values were calculated.

**Table 2:** Sequences of the primers and TaqMan probes.

Gene (GenBank accession)	Oligonucleotide sequence (5′–3′)
*Adipoq* (NM_009605)	
Forward	AAGGAGATGCAGGTCTTCTTGGT
Reverse	CACTGAACGCTGAGCGATACAT
TaqMan Probe	FAM-TGGAATGACAGGAGCTGAAGGGCCA-TAMRA
*Adipor1* (NM_028320)	
Forward	TCATCTACCTCTCCATCGTCTGTGT
Reverse	CAAGCCAAGTCCCAGGAACA
TaqMan Probe	FAM-CATTGTGGCACAGTGGGACCGGTT-TAMRA
*Adipor2* (NM_197985)	
Forward	TTTGCCACCCCTCAGTATCG
Reverse	TGACATAATGCAAGGTAGGGATGAT
TaqMan Probe	FAM-TGTTCGTGGGCTTAGGCCTGAGTGG-TAMRA
*Cd68* (NM_001291058)	
Forward	TACATGGCGGTGGAATACAATG
Reverse	GAACAGCTGGAGAAAGAACTATGCTT
TaqMan Probe	FAM-AGGCAGCACAGTGGACATTCATGGC-TAMRA
*Cybb* (NM_007807)	
Forward	TGTGTCGAAATCTGCTCTCCTTT
Reverse	AAAGTGAGGTTCCTGTCCAGTTGT
TaqMan Probe	FAM-AGTGCGTGTTGCTCGACAAGGAT-TAMRA
*Il1b* (NM_008361)	
Forward	TGACAGTGATGAGAATGACCTGTTC
Reverse	TGATGTGCTGCTGCGAGATT
TaqMan Probe	FAM-ACCCCAAAAGATGAAGGGCTGCTTCC-TAMRA
*Il6* (NM_031168)	
Forward	CGGAGGCTTAATTACACATGTTCTC
Reverse	CAGTTTGGTAGCATCCATCATTTCT
TaqMan Probe	FAM-CGTGGAAATGAGAAAAGAGTTGTGCAATGG-TAMRA
*Nox1* (NM_172203)	
Forward	TTATCGCTCCCAGCAGAAGGT
Reverse	CATGCTAAAGCCTCGCTTCCT
TaqMan Probe	FAM-ATTACCAAGGTTGTCATGCACCCA-TAMRA
*Saa3* (NM_011315)	
Forward	AGTCATCAGCGATGCCAGAGA
Reverse	CCCCACTCATTGGCAAACTG
TaqMan Probe	FAM-CACGGGACATGGAGCAGAGGACTCA-TAMRA
*Spp1* (NM_009263)	
Forward	TTTGCCGTTTGGCATTGC
Reverse	TGGGTGCAGGCTGTAAAGCT
TaqMan Probe	FAM-TCCTCCCTCCCGGTGAAAGT-TAMRA
*Srebf1* (NM_011480)	
Forward	TCCCAAGAGCCCTGCACTT
Reverse	GTCCACAAAGAAACGGTGACCTA
TaqMan Probe	FAM-TTGACACGTTTCTTCCTGAGCAGCGC-TAMRA
*Tnf* (NM_013693)	
Forward	CCAGACCCTCACACTCAGATCAT
Reverse	ACTCCAGCTGCTCCTCCACTT
TaqMan Probe	FAM-CCTGTAGCCCACGTCGTAGCAAACCA-TAMRA

*Adipoq*, adiponectin; *Adipor1*, adiponectin receptor 1; *Adipor2*, adiponectin receptor 2; *Cd68*, macrophage antigen CD68; *Cybb*, superoxide-generating NADPH oxidase heavy chain subunit (Nox2); *Il1b*, interleukin-1β; *Il6*, interleukin-6; *Nox1*, NADPH Oxidase 1; *Saa3*, serum amyloid A3; *Spp1*, secreted phosphoprotein 1 (osteopontin), *Srebf1*, sterol regulatory element binding transcription factor 1; *Tnf*, tumor necrosis factor-α.

### Protein extraction and Western-blot

Similar amounts of EWAT or SCWAT were homogenized and protein content was measured as previously described^65^. Equal amounts of protein (30 μg) were run out in 10% SDS-PAGE, subsequently transferred to nitrocellulose membranes (Bio-Rad Laboratories, Inc., Hercules, CA, USA) and blocked in Tris-buffered saline with Tween 20 (TBS-T) containing 5% nonfat dry milk for 1 h at room temperature (RT). Blots were then incubated overnight at 4 °C with a monoclonal anti-mouse adiponectin mice antibody (MAB3608, Chemicon, Temecula, CA, USA) diluted 1:1,000 in blocking solution. The antigen-antibody complexes were visualized using peroxidase-conjugated anti-rabbit IgG antibody (1:5,000) and the enhanced chemiluminescence ECL detection system (Amersham Biosciences, Buckinghamshire, UK). The intensity of the bands was determined by densitometric analysis with the Gel Doc^TM^ EQ gel documentation system and Quantity One 4·5·0 software for quantitation of images (Bio-Rad)^66^.

### Intrahepatic lipid content

Tissue were homogenized and diluted in saline at a final concentration of 50 mg/mL as previously described^61, 65^. Homogenates were diluted (1:1) in 1% deoxycholate (Sigma-Aldrich) and incubated at 37 °C for 5 min. For triglyceride measurements, samples were diluted 1:100 in the reagent (Infinity™ Triglycerides Liquid Stable Reagent, Thermo Fisher Scientific) and incubated for 30 min at 37 °C. The resulting dye was measured based on its absorbance at 550 nm with a Sunrise ELISA plate reader (Tecan, Männedorf, Switzerland). Concentrations were determined compared with a standard curve of triglycerides (Infinity™ Triglycerides Standard, Thermo Fisher Scientific). The protein content of the preparations was measured by the Bradford method, using BSA (Sigma-Aldrich) as standard. All assays were performed in duplicate.

### Culture of adipocytes and analysis of adiponectin secretion

Mice stromovascular fraction cells (SVFCs) were isolated from EWAT from wt or *ob/ob* mice. Briefly, small pieces (≈500 mg) of adipose tissue were minced and incubated for 1 h at 37 °C with constant shaking in 5 mL of adipocyte medium (DMEM/F-12 [1:1] (Invitrogen, Paisley, UK) supplemented with 16 μmol/L biotin, 18 μmol/L panthotenate, 100 μmol/L ascorbate and antibiotic-antimycotic (Invitrogen)) supplemented with 2% BSA (Sigma-Aldrich) and 2 mg/mL collagenase (Roche). The digestion was stopped adding adipocyte medium supplemented with 10% newborn calf serum (NCS) (Sigma-Aldrich) and suspended cells were then filtered through a 100-μm nylon cell strainer (BD Biosciences, Erembodegem, Belgium) and centrifuged for 10 min at 600 *g*. Adipocytes were decanted and stromovascular pellets were resuspended in adipocyte medium supplemented with 10% NCS. After a filtration through a 70-μm nylon cell strainer (BD Biosciences), cells were spun at 400 *g* for 5 min. SVFCs were resuspended in erythrocyte lysis buffer (0.154 mol/L NH_4_Cl, 10 mmol/L KHCO_3_, 0.1 mmol/L EDTA) and allowed to settle for 10 min at RT, followed by a centrifugation for 10 min at 400 *g*. The cell pellet was resuspended in adipocyte medium supplemented with 10% NCS. SVFCs were grown in six-well plastic plates (5 × 10^5^ cells per well) and maintained at 37 °C in a humidified atmosphere with 95% air-5% CO_2_. Adipocyte differentiation was initiated when cells reached about 80–90% confluence. Cells were stimulated with differentiation medium I (adipocyte medium supplemented with 10% NCS, 0.5 mmol/L 3-isobutyl-1-methylxanthine, 0.1 μmol/L dexamethasone and 10 μg/mL insulin) for 2 days. Cells were then switched to differentiation medium II (adipocyte medium supplemented with 10% NCS and 10 μg/mL insulin) for another 6 days and media were changed every 2 days. Adipocytes were 70–75% differentiated (as determined by morphology) in the 8^th^ day of differentiation. Adipocytes were incubated for 2 h in serum-free adipocyte medium and, then, stimulated with mouse recombinant leptin (PeproTech) at a concentration of 10^−8^ mol/L for 48 h. One sample per experiment was used to obtain control responses in the presence of solvent. Concentrations of adiponectin in the media were measured by ELISA (Biovendor).

### Statistical analysis

Data are presented as mean ± SEM and differences between groups of mice were analyzed by one-way ANOVA followed by Fisher’s LSD tests. The effect of group/treatment in cell cultures was analyzed by unpaired Student’s *t* tests. Correlation between two variables were computed by Pearson’s correlation coefficients (*r*). A *P* value lower than 0.05 was considered statistically significant. The calculations were performed using SPSS 23 (SPSS, Chicago, IL, USA) and GraphPad Prism 6 (GraphPad Software, Inc., La Jolla, CA, USA).
